# Confirmation of the cardiac safety of nolasiban in a randomised cohort of healthy female volunteers

**DOI:** 10.1038/s41598-021-85650-3

**Published:** 2021-03-18

**Authors:** Jörg Täubel, Ulrike Lorch, Christopher S. Spencer, Anne Freier, Dorothée Camilleri, Dilshat Djumanov, Georg Ferber, Line Marchand, Jean-Pierre Gotteland, Oliver Pohl

**Affiliations:** 1 Richmond Pharmacology Ltd., St George’s University of London, Cranmer Terrace, London, SW17 0RE UK; 2 Cardiovascular and Cell Sciences Research Institute, St George’s University of London, London, UK; 3 Richmond Research Institute, St George’s University of London, London, UK; 4 Statistik Georg Ferber GmbH, Riehen, Switzerland; 5 ObsEva SA, Geneva, Switzerland

**Keywords:** Drug discovery, Cardiology, Medical research

## Abstract

Nolasiban is an orally active oxytocin receptor antagonist being developed to increase the efficiency of assisted reproductive technologies. This study evaluated the pharmacokinetics, pharmacodynamics, and cardiac safety of nolasiban in 45 healthy women of child-bearing age. Nolasiban was administered in a fasted state with a standardised lunch served 4.5 h post-dose. Concentration-effect modelling was used to assess the effect of two dosages of nolasiban (900 mg and 1800 mg) on QTc following single-dose administration. We found no significant change in QTc at all tested dosages. Two-sided 90% confidence intervals of geometric mean C_max_ for estimated QTc effects of nolasiban were below the threshold of regulatory concern. The sensitivity of the assay to detect small changes in QTc was confirmed by a significant shortening of QTc between 2 and 4 h after consumption of a meal, which served to validate the model. Independent of the nolasiban assessment, this study also explored the effects of sex hormones on ECG parameters, especially QT subintervals. We found a significant relationship between JTpc and oestradiol. Heart rate was negatively correlated with progesterone. This study confirms the cardiovascular safety of nolasiban and describes relationships of sex hormones and ECG parameters.

## Introduction

Infertility impacts approximately 15% of couples worldwide causing social, economic and psychological burden to those affected. Despite the use of assisted reproductive technologies (ARTs) including in vitro fertilisation (IVF) steadily increasing^1^, the rate of effectiveness of such treatments is around 30% per treatment course^2^.

Successful implementation of the embryo following IVF treatment is a critical stage in achieving pregnancy. The most important predictors for success of embryo transfer are the transfer technique^3^, embryo quality, and uterine receptivity^4^. It has been hypothesised that antagonism of oxytocin receptors (OTRs) could enhance uterine receptivity possibly by decreasing uterine contractions^5^. The rate of uterine contractions at the time of embryo transfer (ET) was shown to be negatively correlated with pregnancy rates after IVF in fresh and frozen thaw cycles^6,7^. Also, endometrial blood flow appears to be a predictive parameter for endometrial receptivity^8,9^ and it has been hypothesised that antagonism of oxytocin receptors could enhance uterine receptivity by improving endometrial perfusion^10^.

**Figure 1:**
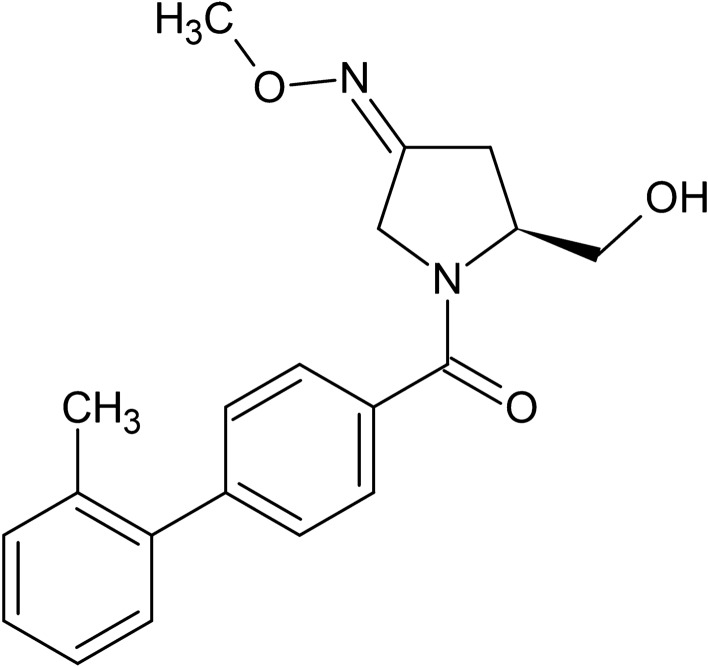
Chemical structure of nolasiban.

Nolasiban (Fig. 1) is a potent, orally-administered, selective OTR antagonist which is developed by ObsEva SA, Switzerland. It has been shown to have the potential to enhance the receptivity of the endometrium to embryo implantation^11^ and thus could increase efficiency of ARTs. Nolasiban is a competitive and reversible OTR antagonist with a K_i_ = 52 nM, and selectivity over vasopressin receptors V1a, V1b and V2^11^. It has been found to inhibit spontaneous and oxytocin-induced uterine contractions in in vivo^11^ and in vitro models^12^.

Determining the cardiovascular effects profile of candidate drugs is an important part of safety assessments in drug development since cardiac toxicity is among the reasons leading drugs to fail during development^13^. Non-clinical cardiovascular safety studies have previously demonstrated a lack of non-clinical evidence of QT/QTc interval prolongation or pro-arrhythmic potential of nolasiban, and available clinical safety data in ART patients dosed up to 900 mg also showed no trend towards a pro-arrhythmic potential or cardiovascular risk associated with nolasiban. Nevertheless, because the OTR is expressed in the heart^14,15^, it is conceivable that application of an OTR antagonist may have a direct effect on the heart. Indeed, oxytocin signalling has been implicated in negative chronotropy and parasympathetic neuromodulation^16^, and oxytocin itself has been observed to have an effect on the QT interval^17,18^. It has previously been established that an oxytocin bolus induces a large and transient QTc interval prolongation, suggesting that it may lead to proarrhythmia^19^. It was also shown that oxytocin could cause a dose-dependent bradycardic effect in pregnant women, however, atosiban (another OTR antagonist) did not modulate heart rate to a clinically significant extent^20,21^. A thorough QT/QTc study with the OTR antagonist retosiban indicated that it had no significant effect on cardiac repolarisation at therapeutic and supratherapeutic doses^22^.

To evaluate the cardiac safety and the pharmacokinetic (PK) profile of single-ascending doses of nolasiban, we performed a phase 1, double-blind, single-centre, randomised, placebo-controlled study. We applied PK and pharmacodynamic (PD) modelling to quantitatively assess cardiac safety^25^. As an exploratory endpoint, this study also evaluated the effect of sex hormone levels on heart rate and QT subintervals. The results of this study are discussed in this paper. At the same time, we collected data to investigate the tolerability and the mechanism of action (MOA) of nolasiban on uterine contractions, perfusion and genomics. Findings from this part of the study are beyond the scope of this article and will be reported separately.

The MOA part of the study required women to synchronize menses and to undergo an artificial hormonal cycle similar to women scheduled for frozen embryo transfer. Hormone (progesterone/oestradiol) measurements and concurrent intensive ECG assessments allowed to investigate the effects of hormonal changes on ECG intervals during the standardized hormonal cycle.

## Materials and methods

### Study design

The present study (EudraCT 2018-003702-36) was a two-part, phase 1, double-blind, single‐centre, randomised, placebo‐controlled study conducted at Richmond Pharmacology, London, UK. Part I aimed to assess the safety, tolerability, and PKs of single‐ascending oral doses of nolasiban. Part II of the study investigated the MOA of nolasiban. All parts were conducted in healthy adult female volunteers (N = 57). Cardiac safety data presented herein was measured in cohorts from part II.

**Figure 2:**
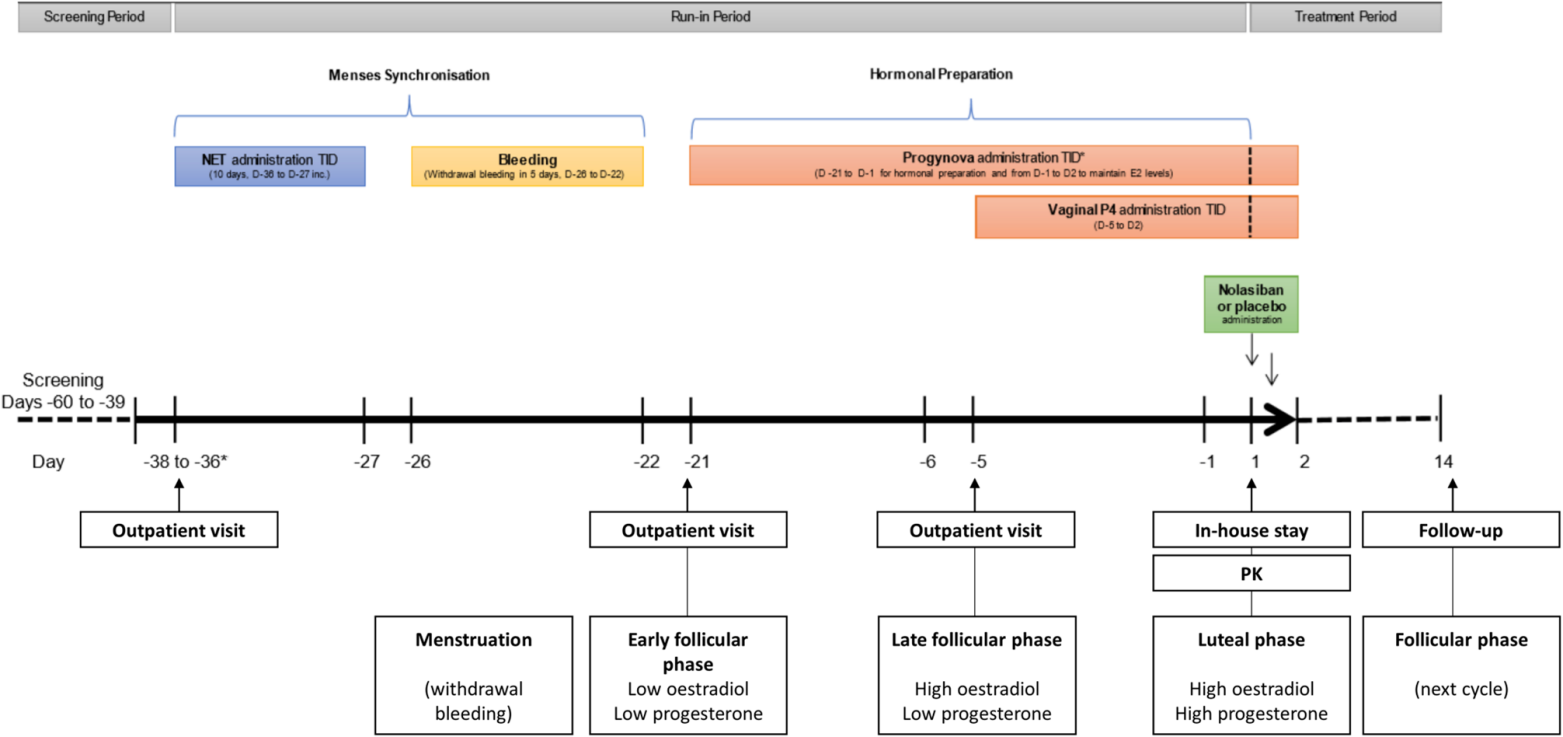
Diagram of synchronised run-in period. Each study day except for Day 1 includes an outpatient visit where a meal was served prior to ECG screening. NET—norethisterone, PK—pharmacokinetics, TID—three times a day. *Outpatient visit was carried out from Day − 38 to Day − 36, NET self-administration began on Day − 36 for all volunteers.

For part II of the study, 159 female volunteers were screened within 22 days prior to the run-in period on Day − 38 (Days − 60 to − 39). Run-in consisted of menstrual synchronisation with norethisterone 5 mg, three times a day, from Day − 36 until Day − 27 (Fig. 2). Upon confirmed withdrawal bleeding, women were pre-treated with an identical hormonal preparation as that used for women undergoing frozen-thawed ET (Oestradiol valerate 2 mg, TID from Day − 21 to Day 2 and vaginal progesterone 200 mg, TID from Day − 5 to Day 2).

**Figure 3:**
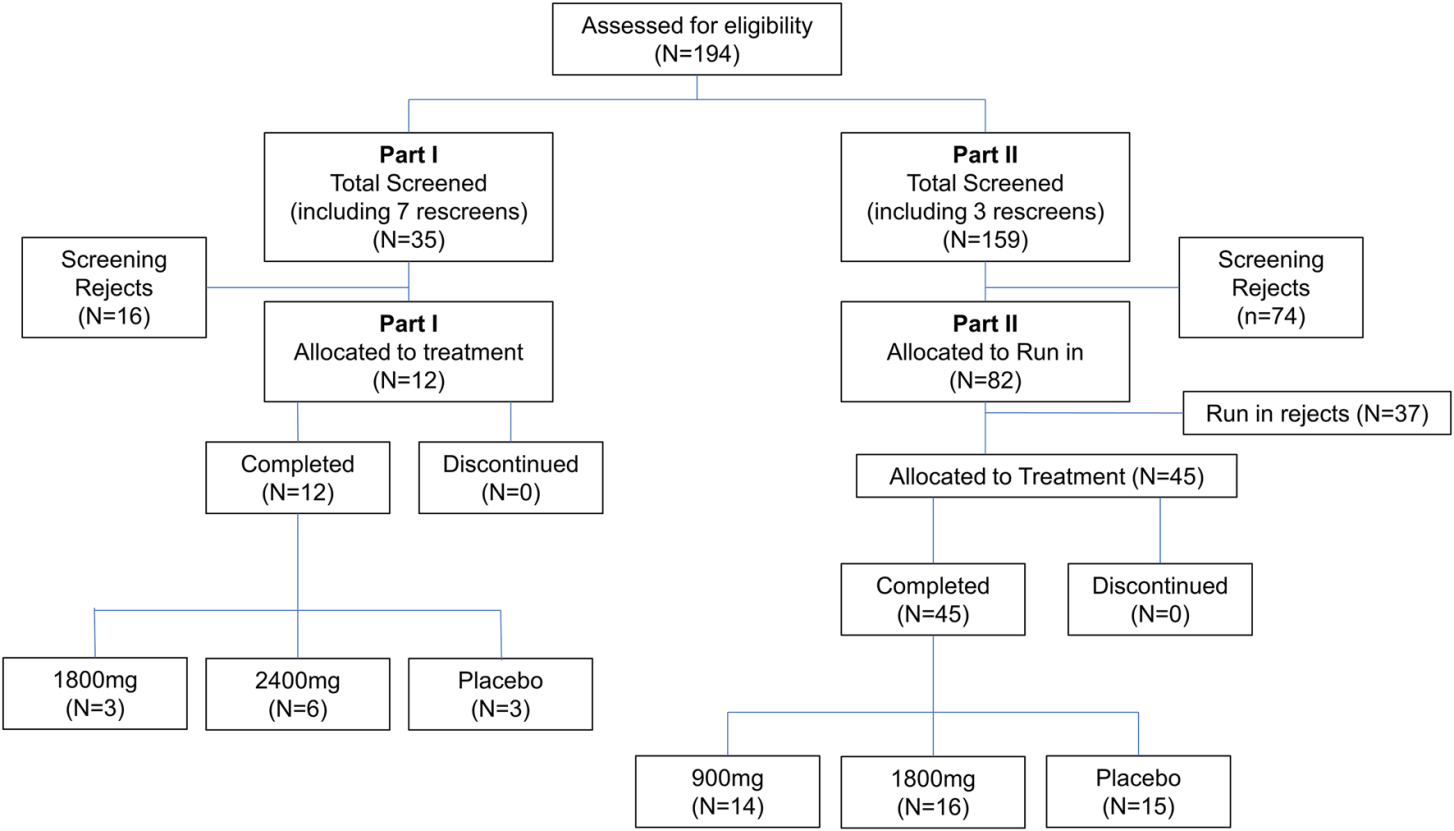
Consort diagram. Cardiac safety of nolasiban was assessed during part II of this study. The sample size in part I did not allow for meaningful statistical analysis, therefore, only data from part II was used during the cardiac safety assessment.

Participants were admitted to the study unit on Day − 1. A single dose of nolasiban (or placebo) was administered on Day 1. Participants remained in the study unit for PK/PD and safety assessments on Day 2 and were discharged from the unit on Day 2 in the absence of any safety concerns. All volunteers attended the unit for a follow-up on Day 14. A total of 45 volunteers were randomised into two cohorts: cohort 1 (nolasiban 900 mg/matching placebo; N = 21) and cohort 2 (nolasiban 1800 mg/matching placebo; N = 21). In each cohort, 14 volunteers received a single dose of nolasiban and seven individuals received a single dose of a matching placebo. Three individuals were assigned into cohort 3 (nolasiban 1800 mg, where two volunteers received a single dose of nolasiban and one received a single dose of a matching placebo). Cohort 3 was an optional cohort included as an adaptive design feature, and was included to ensure enough data was collected for all aspects of the study (Fig. 3).

The study protocol was reviewed and approved by a National Health Service Research Ethics Committee (South Central‐Berkshire B, UK) and the Medicines and Healthcare products Regulatory Authority (London, UK). The study was conducted according to the ethical principles enshrined in UK law, the Declaration of Helsinki, and Good Clinical Practice guidelines.

### PK assessments

Blood samples for plasma PK assessments were collected at 0.5 h pre-dose and at 0.5, 1, 2, 3, 4, 5, 6, 7, 8, 10, 12, and 24 h post-dose. Plasma samples for determination of nolasiban concentrations were analysed by SGS CEPHAC (Saint Benoıt, France), using a validated liquid chromatography–tandem mass spectrometry method with liquid–liquid extraction. Pharmacokinetic assessment was performed using non-compartmental methods similar to those used by Pohl et al. (2015)^21^.

### Cardiac assessments

Intensive cardiac assessments and analysis of drug and hormone-related changes in QT/QTc interval relative to plasma nolasiban concentrations were performed on Day 1 to evaluate the proarrhythmic risk posed by exposure to nolasiban, and on Days − 21, − 5, 1 and 14 to examine the relationship between sex hormones and QT/QTc. All ECG recordings were obtained in triplicate performed at one-minute intervals over three minutes for each time point to confirm accuracy and precision of measurements. ECGs used for this analysis required adjudication by qualified cardiologists in line with the principles set out in the International Conference on Harmonisation (ICH) E14 guideline^23^. The effects of a meal on the ECG were used to establish assay sensitivity^25–28^.

Twelve lead ECGs were recorded using a GE Marquette MAC1200 machine (GE Healthcare, US) and stored on the Medical MUSE information system (GE Healthcare, US). Only those ECGs recorded at a stable heart rate were valid for QT-interval measurements. To investigate the cardiac effect of nolasiban ECG recordings were collected on Day 1, at 2.5, 1, and 0.5 h pre-dose and at 1, 2, 3, 4, 5, 6, 7, 8, 10, 12, and 24 h post-dose. On Days − 21, − 5 and 14, ECGs were recorded after a meal. All ECGs were recorded when volunteers had been resting and were awake in a supine position for at least 10 min, avoiding postural changes.

ECG data files contained ECG data and the result of automated ECG analysis performed by the Marquette 12 SLTM ECG Analysis Program (GE Healthcare, US). All ECGs and their associated automated interval measurements were subsequently blinded before review by a qualified cardiologist to conform with ICH guidance^23^ prior to being used in further statistical analysis. The cardiologist assessed uncorrected QT interval (start of Q wave to end of T wave), the RR interval (start of R wave, to start of next R wave; from which the heart rate was derived using the formula: heart rate = 60,000/RR), PR interval (end of P wave to start of the QRS complex), QRS duration (start of Q wave to end of S wave), subinterval durations, presence or absence of U waves, and ECG variations (Supplement, Figure S1). The cardiologist’s experience with manual on-screen overreading using electronic calipers in MUSE was used to correct implausible readings presented by the automated process. All ECGs were overread by the same cardiologist who was blinded to both treatment and the time point of the recording. If manual adjustments were deemed necessary, and the first overreader requested adjudication, a second cardiologist performed overreading and assessment. Likewise, if the second cardiologist requested adjudication, a third cardiologist performed overreading and assessment. The QT interval was corrected for heart rate used using Fridericia’s formula (QTcF)^29^. A total of 3215 ECGs were used to calculate 1030 triplicate means after adjudication. The average was taken of the three pre-dose values for use as a baseline.

### Statistical analysis

Statistical analyses were based on QTcF and all data from Day 1. All participants with valid ECG data for pre-dose baseline and the time points on Day 1 were included in the QTcF analysis set.

A concentration-effect analysis was chosen as the primary analysis to correlate heart rate-corrected QT interval duration with exposure to nolasiban. A linear model with random effects for both concentration and intercept became singular and was therefore discarded. Convergence was given for a model with a random intercept only. The model had a centred baseline (i.e. baseline minus mean of baseline for all volunteers) as covariate and time and treatment as discrete factors. Based on this model, predictions of the effect at the geometric mean of the nolasiban C_max_ were made for each dose group (90% confidence interval (CI)).

To confirm the validity of a linear concentration-effect model, presence or absence of hysteresis was confirmed visually by comparing QTcF changes over time versus the PK profile. A change in heart rate upon exposure to nolasiban was assumed to not be concentration-dependent if the difference to placebo was below 10 bpm for all Day 1 time points. A predefined, validated test for assay sensitivity was specified in the statistical analysis plan.

### Estimation of the sex hormone effect on cardiac parameters

Concentration-effect modelling was conducted to explore the effects of progesterone and oestradiol separately on heart rate, and each of the subintervals; QTcF, QRS, JTpc (end of P wave to peak of T wave), and TpTe (start of T wave to end of T wave), (Supplement, Figure S1). A substantial effect of hormone levels on heart rate did not exceed 10 bpm at any point, thus, standard heart rate correction methods were applied to QTcF and JTpc intervals. Levels of oestradiol and progesterone were measured in blood drawn on Days − 21, − 5, 1 (− 0.5 h), Day 2 (24 h), and Day 14. Baseline data was set to the pre-dose average on Day 1. Only drug-free data were used, therefore, Day 14 was excluded. Primary models for each cardiac parameter were those with the lowest Akaike Information Criterion (AIC) score—as a measure of the fit of the model.

Linear mixed effects models were fitted to the ECG analysis data and the effect on heart rate and cardiac subintervals was estimated using the oestradiol-change-from-baseline and the progesterone-change-from-baseline as parameters. Models including neither hormone parameter, both hormone parameters, and each separately were investigated, and the AIC score was used as the criterion for model selection. Post-hoc analyses were conducted incorporating baseline and Day only as explanatory variables.

## Results

### Study participants

Healthy women of reproductive age (20–37 years), weighing between 50.8 kg and 85.5 kg, and with a body mass index between 16.5 and 29.9 kg/m^2^ were recruited for the study. Participants were excluded if they presented with a disease or disorder which could influence the study result or put them at risk. Exclusion criteria extended to significant abnormalities in the rhythm, conduction or morphology of resting electrocardiogram (ECG) or abnormalities interfering with the interpretation of QTc interval changes. All participants provided written informed consent and completed the study as described in the protocol. Demographic data and volunteer disposition are shown in Table 1.

**Table 1:** Volunteer demographics.

			900 mg (N = 14)	1800 mg (N = 16)	Placebo (N = 15)	Total (N = 45)
Age (years)		Mean (± SD)	28.9 (± 4.0)	28.4 (± 6.6)	27.7 (± 5.6)	28.3 (± 5.4)
		Range	20–35	20–37	20–37	20–37
Height (cm)		Mean (± SD)	166.7 (± 6.3)	164.4 (± 6.3)	164.5 (± 8.2)	165.2 (± 6.9)
		Range	153–177	157–177	149–184	149–184
Body Mass Index (kg/m^2^)		Mean (± SD)	25.28 (± 2.76)	23.38 (± 2.35)	24.57 (± 2.54)	24.37 (± 2.61)
		Range	20.4–29.1	19.5–28.1	21.1–29.9	19.5–29.9
Race	Asian	n (%)	1 (7.1)	2 (12.5)	0	3 (6.7)
	Black/African	n (%)	6 (42.9)	4 (25.0)	3 (20.0)	13 (28.9)
	White/Caucasian	n (%)	6 (42.9)	7 (43.8)	11 (73.3)	24 (53.3)
	Other	n (%)	1 (7.1)	3 (18.8)	1 (6.7)	5 (11.1)
Ethnicity	Non-Hispanic	n (%)	14 (100.0)	13 (81.3)	14 (93.3)	41 (91.1)
	Hispanic	n (%)	0	3 (18.8)	1 (6.7)	4 (8.9)

*SD* standard deviation.

Breakfast served on Day 1 was prepared according to US Food and Drug Administration (FDA) standards^24^. It contained 666 kcal with a ratio of 69% carbohydrate to 10% fat to 21% protein.

### Pharmacokinetics

Following a single oral dose of 900 mg nolasiban, plasma concentrations were detectable from the first sampling time point (0.5 h) in all volunteers, and T_max_ occurred between 2 and 8 h post-dose in all volunteers (Supplement, Figure S2).

Pharmacokinetic parameters are shown in Table 2. Mean C_max_ and AUC_0–24 h_ values in individuals receiving a 1800 mg dose were roughly double of those receiving a 900 mg dose. Median T_max_ values for both doses were roughly the same. Secondary or multiple peaks were observed in all participants in both dosages between 6 and 8 h. The start of the terminal disposition phase for both doses occurred at around 12 h. The study design did not foresee pharmacokinetic sampling beyond 24 h and the resulting proportion of the extrapolated AUC_0-inf_ was 40.34% for 900 mg and 45.07% for 1800 mg which did not allow for a reliable estimate of the elimination rate constant and associated PK parameters.

**Table 2:** Pharmacokinetic parameters following dosing with nolasiban.

Nolasiban dosage, mg	Mean C_max_, ng/mL (± SD)	Maximum individual C_max_, ng/mL (± SD)	Median T_max_ (hours)	AUC_0–24 h_, ng/mL (± SD)
900	3754.6 ± 815.9	6080	3.9	45,418.8 ± 7957.9
1800	7692.0 ± 1640.8	10,500	4.0	103,718.4 ± 18,990.8

### Cardiac assessments

Primary analysis was conducted as described previously^26^ using the change from average baseline of QT interval corrected by Fridericia’s formula^29^. The time course of all ECG parameters was described by summary statistics.

**Figure 4:**
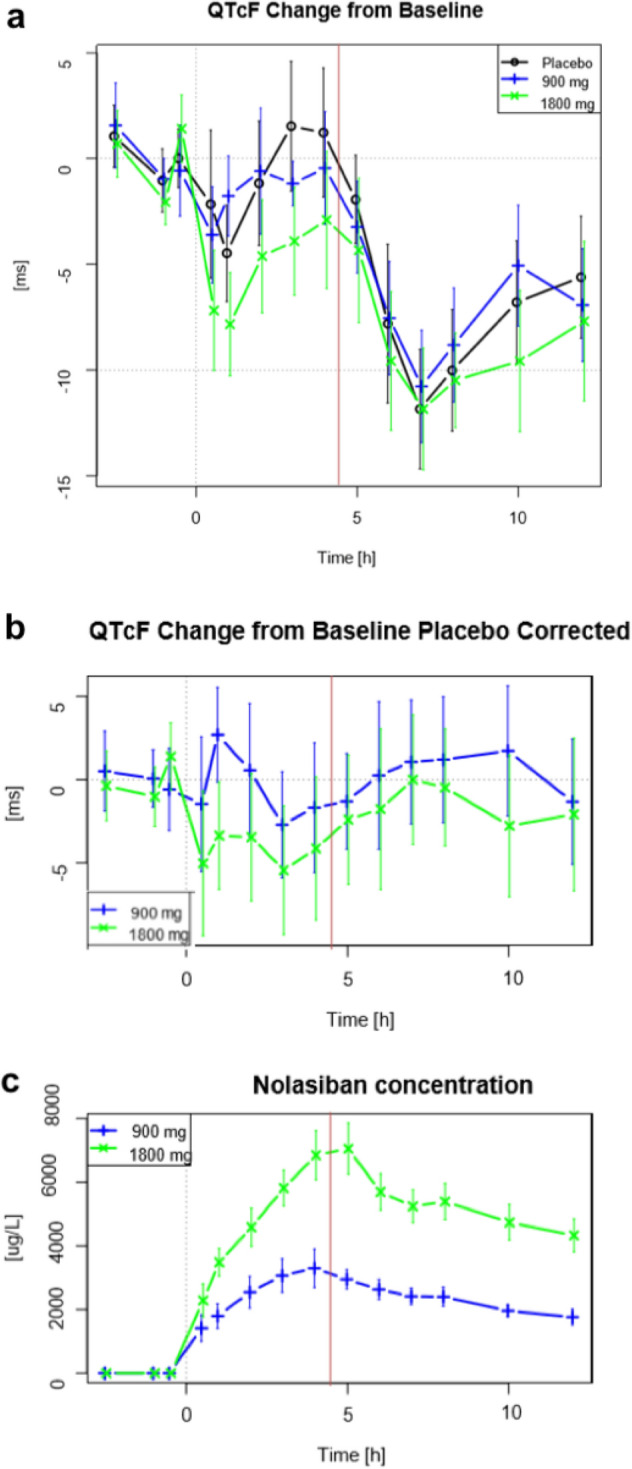
(**a**) Change from baseline QTcF, (**b**) Change from baseline QTcF—difference to placebo, (**c**) nolasiban concentration. Baseline is the average of mean values from pre-dose time points on Day 1. Plotted are mean values and 90% confidence intervals. The red line indicates provision of a meal, given 4.5 h post-dose.

All volunteers in part II of the study were included in the primary analysis set. One triplicate at the two-hour time point in the 1800 mg cohort was excluded because it was outside the proper time window. The time course of QTcF was dominated by two effects: a small shortening of 5 ms immediately after drug administration, and a substantial post-prandial shortening of 10 ms (Figs. 4a,b). There were no clinically significant changes in ECG morphology and mean heart rate, QRS duration, QRS axis, QT interval or RR interval. There were no other notable changes in mean values for any other ECG parameter in any cohort over time. Maximal mean values for QTc for both doses and placebo were observed at pre-dose, and minimal mean values were detected between 6 and 8 h.

Heart rate showed a marked post-prandial increase of up to 10 bpm, which was independent from the treatment group. The mean change in heart rate from baseline corrected for placebo was up to 4.3 bpm at 5 h, indicating that nolasiban has a marginal dose-dependent effect on heart rate. However, a statistically significant effect of nolasiban on heart rate was excluded based on the one-sided 5% level criteria.

### Model-based concentration-effect analyses

In order to identify any PK/PD hysteresis—herein defined as the delay between drug effect on QTcF and plasma drug concentration—the concentration of nolasiban and ΔΔQTcF for each dose was plotted against time (Fig. 4). The plots do not show any indication of PK/PD hysteresis. Specifically, there was no indication of a delayed effect on QTcF, so hysteresis was excluded.

The model showed a non-significant, negative effect of nolasiban on ΔQTcF. There was no significant treatment intercept, therefore, the model was adopted as primary. The estimates of slope and treatment intercept were both negative (Supplement, Figure S3) and predicted effects of nolasiban at geometric mean concentrations seen in the dose groups were also negative. The upper limits of the 90% confidence intervals for the predictions were all below 1 ms (Table 3).

**Table 3:** Predicted effects of nolasiban on ΔQTcF at geometric mean maximum concentrations for each dose group.

Model	AIC	Residual error	Dose (mg)	C_max_ (ug/L)	Estimate (ms)	SE	df	t-value	90% confidence interval	
Primary	2816.5	4.7	900	3651	− 1.9	1.32	Inf	− 1.40	− 4.0	0.3
			1800	7521	− 3.0	1.58	Inf	− 1.92	− 5.6	− 0.4

*SE* standard error, *df* degrees of freedom.

### Sensitivity of the assay

The sensitivity of the experiment to consistently detect changes in QTcF was assessed by measuring the effect of ingesting a standard meal post-dose. A balanced lunch was served at 4.5 h post-dose, and the effect on QTcF was examined 2 to 4 h later. The time course of the food effect was estimated from the estimates of time effect in the primary model. Compared with the average of the value before the meal (3 h and 4 h), the QTcF consistently dropped at 2 to 4 h after the meal (6, 7, and 8 h). Estimated changes are shown in Fig. 4a. There was a QTcF-shortening of between 7.5 and 11 ms, and the 90% confidence intervals were well below 5 ms. These results confirm that the experiment is sensitive enough to detect changes in QTcF.

**Figure 5:**
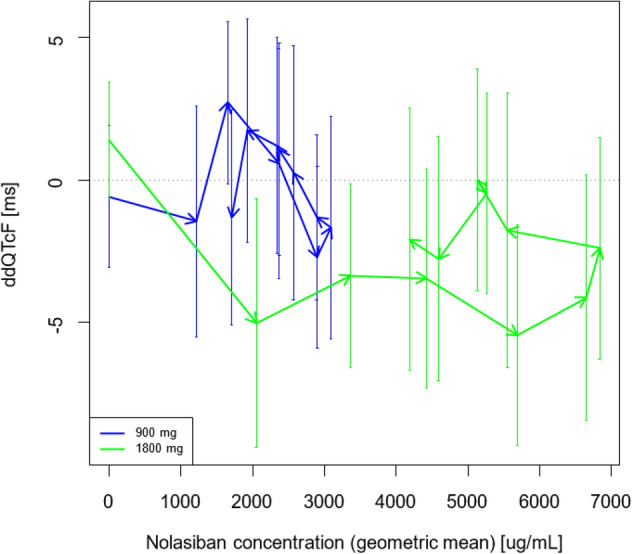
Hysteresis loops for 900 mg and 1800 mg nolasiban doses. There is no indication of any concentration related effect, and particularly not a delayed effect.

Figure 5 shows hysteresis loops for the two highest dose groups. There is no indication of any concentration related effect, and in particular not of a delayed one. For the higher dose group, the loop is anti-clockwise, which, in combination with a negative effect, would indicate proteresis. Exposure-normalised Glomb-Ring Index values for 900 mg and 1800 mg doses were calculated to be 0.002 and 2.1 respectively, supporting the observations in the plotted loops.

### Concentration effect of sex hormones on heart rate and cardiac subintervals

Beyond the effect of nolasiban on cardiovascular safety, we studied the impact of oestradiol and progesterone on ECG intervals. When observing basic changes in oestradiol and progesterone levels, we found no clinically significant or unexplained trends, and the two hormones cycled as expected (see Supplement, Figure S4, Table S1). Modelling indicated that progesterone and oestradiol concentration had no effect QRS or TpTe. Modelling showed that oestradiol concentration had a small but insignificant effect on QTcF duration (0.013 ms per ng/L). Progesterone concentration was found to have a significant, negative effect on heart rate (− 0.158 per nmol/L). Modelling also indicated that oestradiol concentration had a significant, positive effect on JTpc (0.02 ms per ng/L) (Table 4).

**Table 4:** Significant estimates of fixed effects of sex hormones on cardiac parameters.

Parameter	Model	AIC	Effect	Effect estimate	SE	d.f	T value	90% CI	
Heart rate	Progesterone, without baseline	1065.8	Progesterbone (ms per nmol/L)	− 0.158	0.0218	Inf	− 7.23	− 0.194	− 0.122
			Intercept (ms)	0.5	0.82	Inf	0.59	− 0.9	1.8
QTcF	Oestradiol without baseline	1225.5	Oestradiol (ms per ng/L)	0.013	0.0080	Inf	1.58	− 0.001	0.026
			Intercept (ms)	1.3	0.91	Inf	1.41	− 0.2	2.8
QRS	None	726.5	Intercept (ms)	0.5	0.21	Inf	2.28	0.1	0.8
JTpc	Oestradiol without baseline	1144.8	Oestradiol (ms per ng/L)	0.020	0.0060	Inf	3.41	0.011	0.030
			Intercept (ms)	0.7	0.85	Inf	0.81	− 0.7	2.1
TpTe	Baseline only	940.5	Baseline (ms per ms)	− 0.16	0.056	Inf	− 3.19	− 0.27	0.09
			Intercept (ms)	− 0.1	0.39	Inf	− 0.25	− 0.7	0.5

*SE* standard error, *df* degrees of freedom.

In order to understand whether the effects of progesterone and oestradiol were the variables driving the variation observed in cardiac parameters, ad hoc statistical analyses were applied using ‘day of the month’ only as an effect estimate. The AIC scores for the heart rate models were 1042.4 and 1039.2 with and without baseline, respectively; AIC scores for JTpc were 1133.5 and 1129.1 with and without baseline, respectively (Supplement, Table S2). These scores were all lower than the primary models described above, indicating that ‘day of the month’ models fit the data better than hormone models.

## Discussion

Our findings demonstrate that nolasiban did not lengthen the QTcF interval, indicating that it did not inhibit cardiac repolarisation in a population of healthy females of childbearing age at doses of 900 mg and 1800 mg. Cardiac safety was examined during part II of the study because of the greater cohort size (part I: N = 12; part II: N = 45), thus providing a larger sample size for relevant dosages. This is consistent with existing non-clinical or clinical cardiovascular safety data of nolasiban which did not point to any potential prolongation of the QT/QTc interval (ObsEva, data available on request).

The food effect validated the assay, demonstrating a QTcF shortening of 7.5 to 11 ms. This shortening, and its 90% confidence intervals were larger than 5 ms, in line with previous observation^26,30^, confirming that the study was able to detect small changes in QTc. Our findings provide further evidence that the QT interval reduction response to a standardised meal can be a useful and reproducible means to confirm the sensitivity of an assay, making it a viable alternative for moxifloxacin, as previously shown^26,31^. A standardised meal is a non-pharmacological, non-toxic positive control in QT studies, and because it shortens rather than lengthens the QT interval, it is a valuable method for studying groups at risk of QT-prolongation.

It is particularly noteworthy that the effect of a meal was seen despite a mild shortening effect of nolasiban. This suggests that different mechanisms may be driving the shortening effect of nolasiban versus that of a meal. Previous studies have indicated that an oxytocin bolus has a QTc-prolonging effect^19^. It is, therefore, coherent that blockade of the OTR via an antagonist like nolasiban will have a QTc-shortening effect. A similar effect has been observed with the OTR antagonist retosiban, where a small shortening effect on QTc was observed (< 5 ms for doses of 100 mg and 800 mg, respectively)^22^. In comparison, the meal effect on QT duration is mediated by the protein C-peptide^32^. Reports of drug-induced QT-interval shortening remain rare as most cardiac safety evaluations focus on QT-prolongation. This is because of the well-documented phenomenon that QT-prolongation increases the risk of Torsades de Points. The proarrhythmic potential of QT-shortening is therefore not well-understood and future studies are needed to determine the long-term effects of QT-shortening drugs on cardiovascular health across diverse patient populations. However, the shortening effect observed in this study was very low (≤ 5 ms at all doses) and is unlikely to pose a proarrhythmic risk^33^.

In parallel, we examined the effects of oestradiol and progesterone over the course of 21 days prior to the start of the study. We found that ECG parameters and hormone levels varied over this synchronisation period. For QTcF, QRS, and TpTe, no significant correlation between hormone level and the duration of the subinterval was observed. For JTpc, a significant relationship to oestradiol was found, which raises the question whether QTc-prolongation in women may be due to the effect of oestradiol on JTpc. It has previously been observed that women of child-bearing potential have a longer QTc interval^34^. Mechanistically, this appears to be coherent with the observation that oestrogen downregulates potassium (I_ks_) ion channels involved in cardiac repolarisation^35^.

Further, we found that heart rate was significantly negatively affected by progesterone levels. Progesterone has previously shown to lower peripheral vascular resistance, which lowers resting peripheral vascular resistance leading to a reduction in cardiac filling pressure and cardiac output^36^. However, the precise effect of progesterone on heart rate remains to be determined, as previous findings have been conflicting^37,38^. One limitation of these findings is that when using cycle phase rather than hormone level as an explanatory variable, the fit of the models was improved. This suggests that the effect on ECG may be led by cycle phase and could depend on additional variables as opposed to oestradiol and progesterone alone.

In conclusion, the findings of this study confirm that nolasiban is not associated with any QTcF prolongation of regulatory concern in healthy women at doses up to 1800 mg. Further, we observed a relationship between the duration of heart rate and progesterone levels, and the JTcp subinterval and oestrogen levels. However, future studies are required to deduce if these are direct or indirect relationships.

## Supplementary Information

Supplementary Information.

## Data Availability

Requests for access to data should be addressed to the corresponding author.
