# Supplementary material for: Confirmation of the cardiac safety of nolasiban in a randomised cohort of healthy female volunteers
Source: Sci Rep. 2021 Mar 18;11:6404. doi: 10.1038/s41598-021-85650-3 (PMC7973531; doi:10.1038/s41598-021-85650-3)
Supplement: Supplementary file 1 — Supplementary Information. [file 41598_2021_85650_MOESM1_ESM.docx]

**Confirmation of the cardiac safety of nolasiban in a randomised cohort of healthy female volunteers – Supplementary Material**

Jörg Täubel^1,2^, Ulrike Lorch^1^, Christopher S. Spencer^3^, Anne Freier^3^, Dorothée Camilleri^1^, Dilshat Djumanov^1^, Georg Ferber^4^, Line Marchand^5^, Jean-Pierre Gotteland^5^, Oliver Pohl^5^

^1^ Richmond Pharmacology Ltd., London, UK

^2^ Cardiovascular and Cell Sciences Research Institute, St George’s University of London, London, UK

^3^ Richmond Research Institute, St George’s University of London, London, UK

^4^ Statistik Georg Ferber GmbH, Riehen, Switzerland

^5^ ObsEva SA, Geneva, Switzerland

**Corresponding author:** Jörg Täubel

Richmond Pharmacology Ltd., St George's University of London, Cranmer Terrace SW17 0RE, London, United Kingdom

E-mail: [j.taubel@richmondpharmacology.com](mailto:j.taubel@richmondpharmacology.com)

**
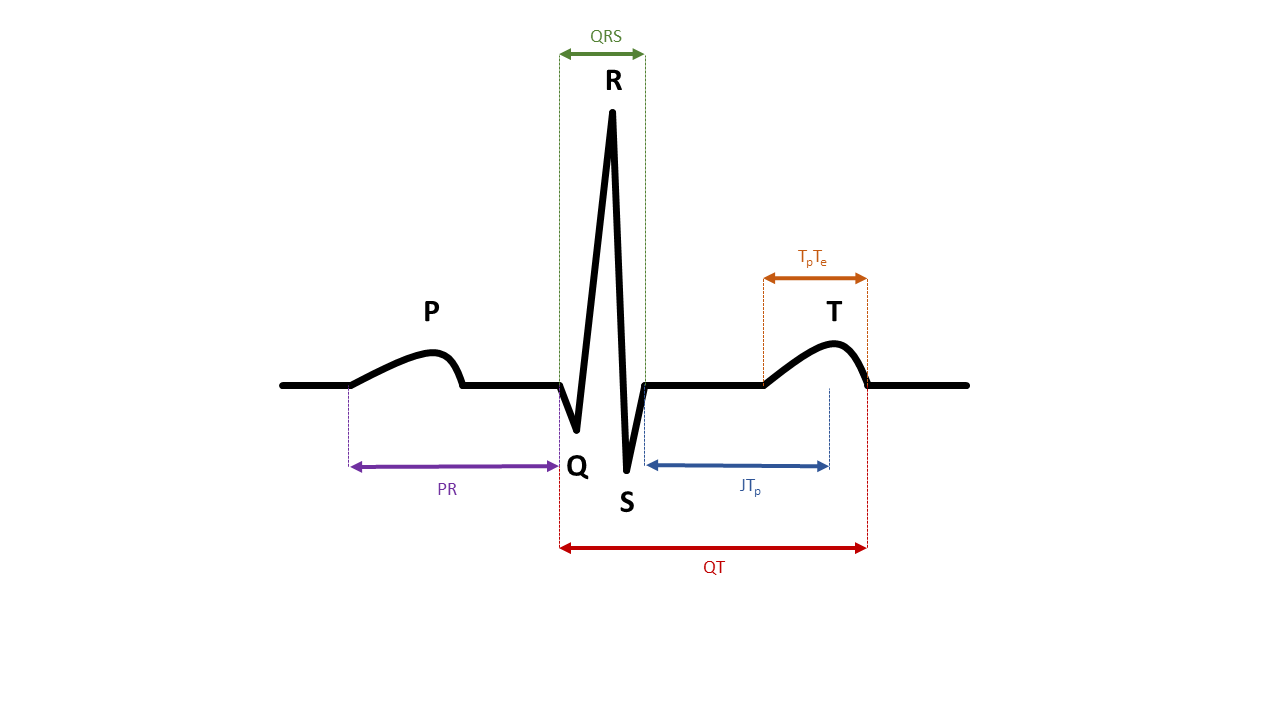
**

**
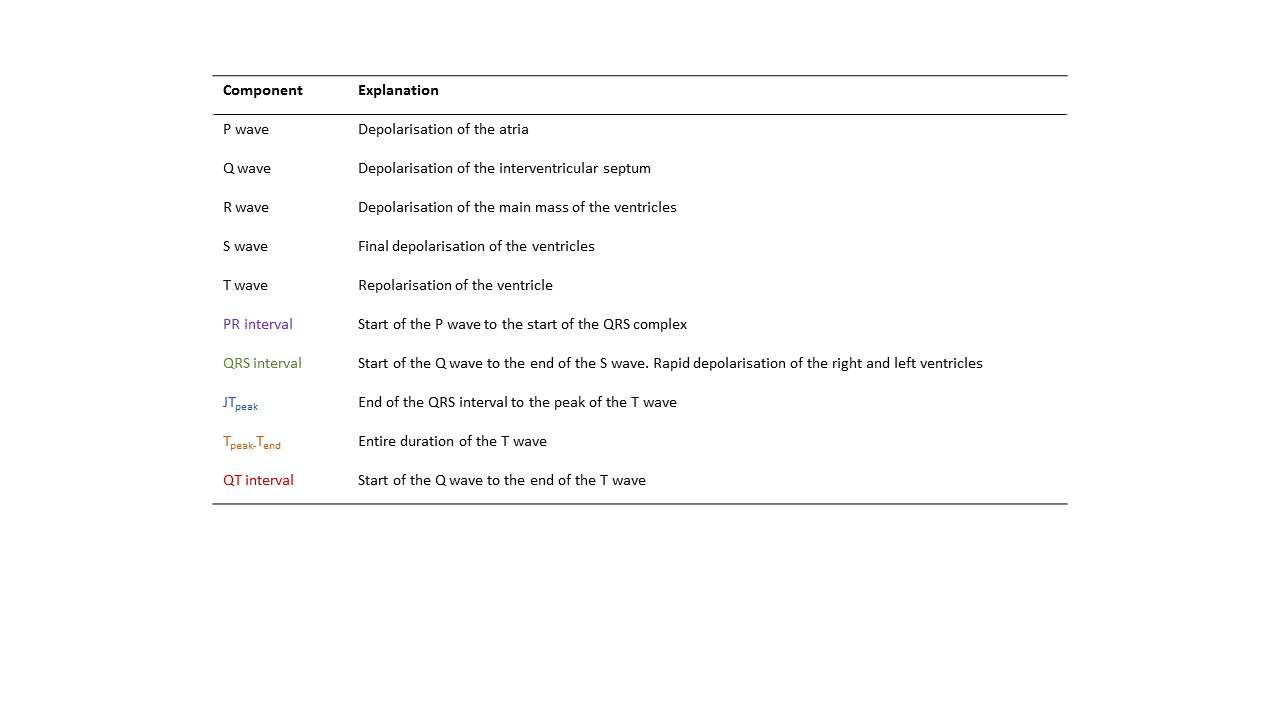
**

**Figure S1.** Diagrammatic representation of cardiac subintervals

**
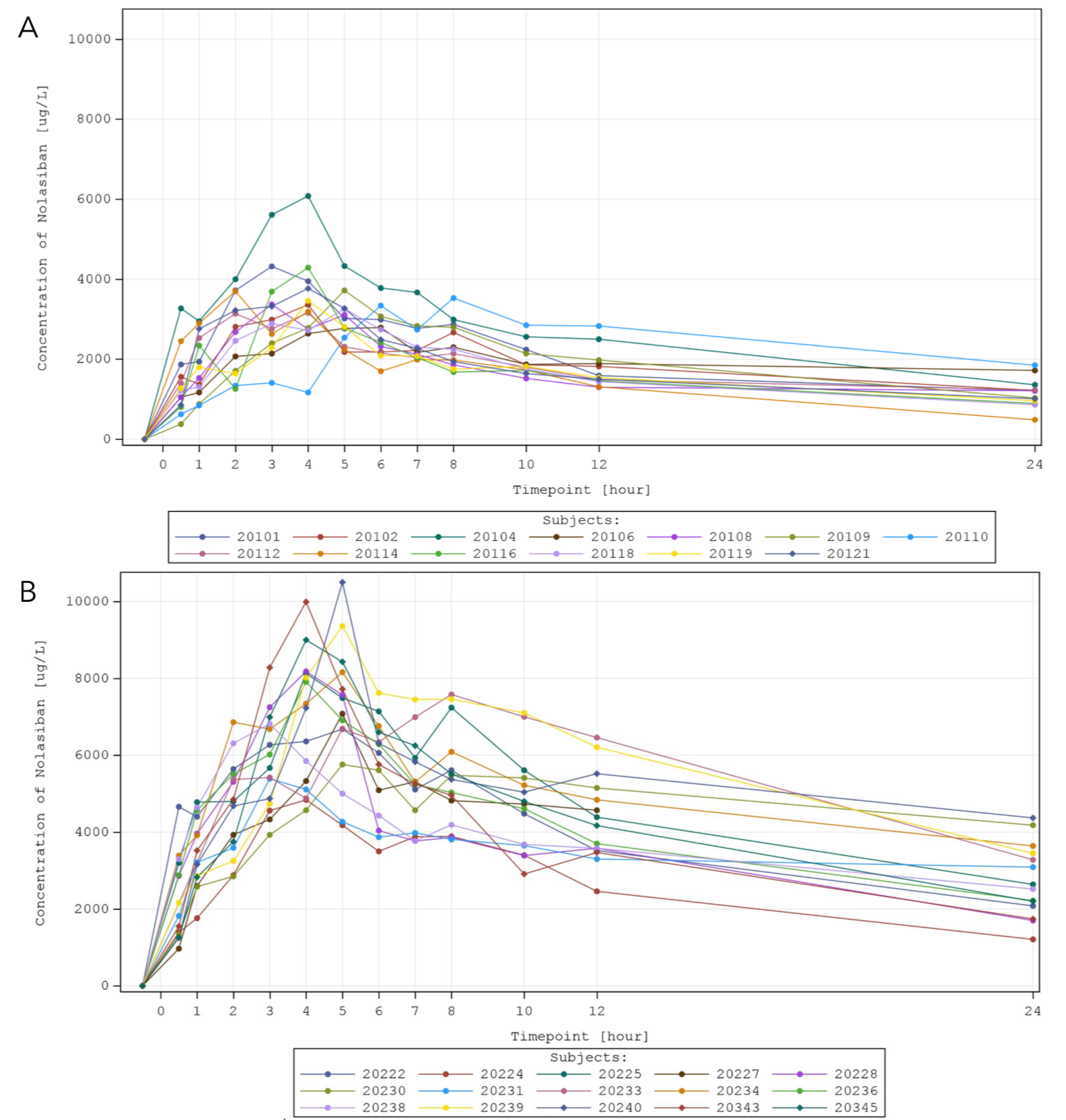
**

**Figure S2.** Time curve for nolasiban concentration in plasma for **A** cohort 1, 900 mg dose, and **B** cohort 2, 1800 mg dose.


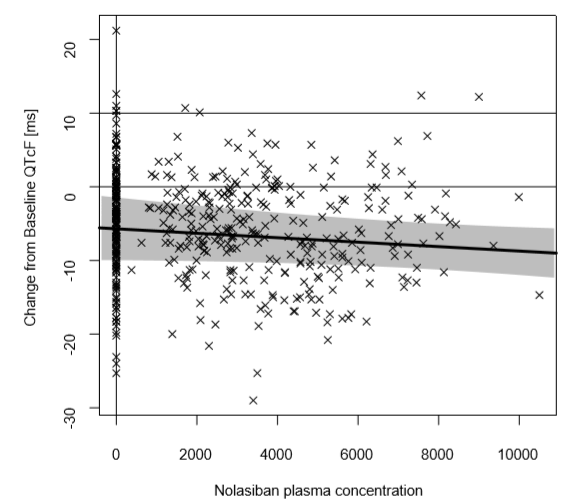


**Figure S2.** Scatter plot of ΔQTcF by concentration of nolasiban. A regression line has been added according to the primary linear model and is adjusted for the mean time effect. The 90% confidence interval range is shown in grey.


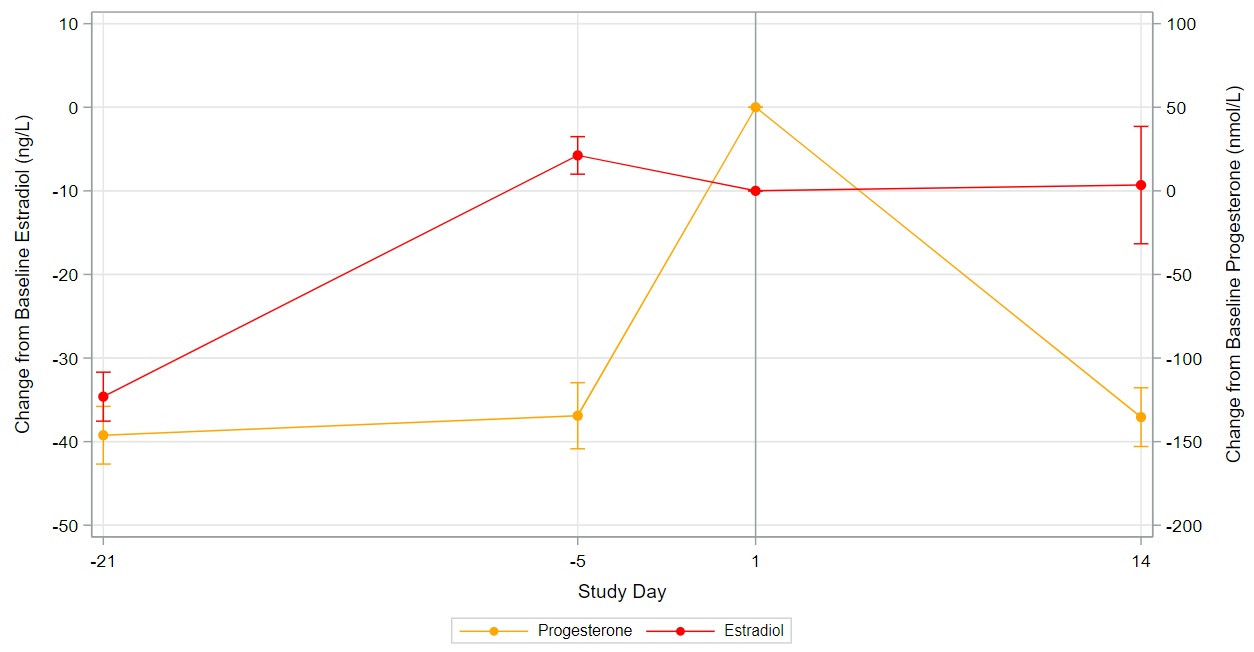


**Figure S3.** Changes from baseline for the hormones, progesterone and estradiol over the study period.

**Table S1.** Change from baseline for ECG parameters according to menstrual phase.

| **Parameter** | **Day** | **Phase** | **Mean** | **Median** | **SD** | **Range** | |
| --- | --- | --- | --- | --- | --- | --- | --- |
| Heart rate | -21 | Menstruation | 7.7 | 7.2 | 6.44 | -6 | 23 |
|  | -5 | Ovulation | 4.7 | 2.8 | 6.16 | -6 | 21 |
|  | 1 | Luteal | -0.4 | -0.2 | 1.97 | -4 | 4 |
|  | 14 | Follicular | 7.9 | 6.8 | 5.71 | -3 | 19 |
| QTcF | -21 | Menstruation | -3.1 | -3.2 | 9.37 | -28 | 21 |
|  | -5 | Ovulation | 0.4 | 2.4 | 8.79 | -25 | 15 |
|  | 1 | Luteal | 1.1 | 0.0 | 7.07 | -12 | 38 |
|  | 14 | Follicular | 5.4 | 4.3 | 9.86 | -13 | 34 |
| QRS | -21 | Menstruation | -0.1 | 0.0 | 1.86 | -4 | 4 |
|  | -5 | Ovulation | 0.6 | 0.7 | 1.86 | -4 | 6 |
|  | 1 | Luteal | -0.1 | 0.0 | 0.57 | -1 | 1 |
|  | 14 | Follicular | 1.5 | 1.0 | 3.03 | -2 | 17 |
| JTpc | -21 | Menstruation | -3.1 | -3.9 | 9.00 | -24 | 13 |
|  | -5 | Ovulation | 0.8 | 1.1 | 8.41 | -33 | 19 |
|  | 1 | Luteal | 0.0 | -0.1 | 3.58 | -11 | 7 |
|  | 14 | Follicular | 2.9 | 1.9 | 7.85 | -14 | 19 |
| TpTe | -21 | Menstruation | -0.7 | -1.3 | 5.07 | -13 | 13 |
|  | -5 | Ovulation | -0.8 | -0.2 | 3.56 | -12 | 5 |
|  | 1 | Luteal | 0.3 | 0.1 | 1.57 | -3 | 4 |
|  | 14 | Follicular | 0.8 | 0.6 | 5.14 | -10 | 13 |

Abbreviations: SD = Standard Deviation

**Table S2.** AIC, residual variability and standard deviation (SD) of random effects of the models investigated.

| **Parameter** | **Model** | **AIC** | **SD of** | |
| --- | --- | --- | --- | --- |
|  |  |  | **Residual** | **Random Intercept** |
| Heart rate | Day with BL | 1042.4 | 4.93 | 2.10 |
|  | Day without BL | 1039.2 | 4.93 | 2.16 |
| QTcF | Day with BL | 1200.6 | 7.83 | 3.91 |
|  | Day without BL | 1197.6 | 7.83 | 4.10 |
| QRS | Day with BL | 716.5 | 1.74 | 1.05 |
|  | Day without BL | 710.7 | 1.74 | 1.03 |
| JTpcJ | Day with BL | 1133.5 | 6.01 | 4.50 |
|  | Day without BL | 1129.1 | 6.01 | 4.52 |
| TpTe | Day with BL | 937.0 | 3.49 | 1.79 |
|  | Day without BL | 940.4 | 3.49 | 2.14 |
